# INCLUDING FEEDBACK FROM OLDER ADULTS WITH VARYING HEALTH CONDITIONS TO INFORM EXERGAME DESIGNS

**DOI:** 10.1093/geroni/igac059.1037

**Published:** 2022-12-20

**Authors:** Breanna Crane, Brittany Drazich, Janiece Taylor, Kyle Moored, Omar Ahmad, John Krakauer, Michelle Carlson

**Affiliations:** Johns Hopkins Bloomberg School of Public Health, Baltimore, Maryland, United States; University of Maryland, Baltimore, Maryland, United States; Johns Hopkins University, Baltimore, Maryland, United States; Johns Hopkins Bloomberg School of Public Health, Baltimore, Maryland, United States; Johns Hopkins University, Baltimore, Maryland, United States; Johns Hopkins University, Baltimore, Maryland, United States; Johns Hopkins Bloomberg School of Public Health, Baltimore, Maryland, United States

## Abstract

Exergames are emerging technologies that combine “exercise” and “video games” to promote enjoyable cognitive and physical activities. Exergame studies often exclude individuals at greatest risk for adverse health outcomes (e.g., oldest-old, living with chronic conditions, comorbidities, or functional limitations). Thus, it is important to 1) understand motivators and barriers for joining and adhering to exergame studies and 2) capture perspectives from individuals commonly excluded from these studies. We conducted three focus groups among 14 older adults (mean age=79±9 years) with varying health conditions who participated in a novel three-dimensional exergame feasibility study. Data were analyzed using the “Sort and Sift, Think and Shift” approach. Motivators for joining were generativity, peer referrals, self-improvement, and curiosity. Motivators for retention were accomplishment, enjoyability, and exercise. Barriers to participation included frustration and pain/fatigue. We also discuss how participants’ feedback influenced future exergame design. Findings will aid in promoting scalable, enjoyable, and accessible exergame interventions for all.

